# Combination of Proactive Molecular Risk Classifier for Endometrial cancer (ProMisE) with sonographic and demographic characteristics in preoperative prediction of recurrence or progression of endometrial cancer

**DOI:** 10.1002/uog.23573

**Published:** 2021-09-01

**Authors:** L. S. E. Eriksson, D. Nastic, P. G. Lindqvist, S. Imboden, H. Järnbert‐Pettersson, J. W. Carlson, E. Epstein

**Affiliations:** ^1^ Department of Pelvic Cancer Karolinska University Hospital Stockholm Sweden; ^2^ Department of Women's and Children's Health Karolinska Institute Stockholm Sweden; ^3^ Department of Pathology and Cytology Karolinska University Hospital Stockholm Sweden; ^4^ Department of Oncology–Pathology Karolinska Institute Stockholm Sweden; ^5^ Department of Obstetrics and Gynecology Södersjukhuset Stockholm Sweden; ^6^ Department of Clinical Science and Education Karolinska Institute, Södersjukhuset Stockholm Sweden; ^7^ University of Bern, Department of Obstetrics and Gynecology University Hospital of Bern Bern Switzerland

**Keywords:** endometrial neoplasm, molecular diagnostics, neoplasm assessment, risk assessment, ultrasonography

## Abstract

**Objective:**

To evaluate the ability of demographic and sonographic variables and the Proactive Molecular Risk Classifier for Endometrial cancer (ProMisE) classification to predict preoperatively tumor recurrence or progression in women with endometrial cancer.

**Methods:**

The study included 339 women with histologically confirmed endometrial cancer who underwent expert transvaginal ultrasound in a single center before surgery as part of the prospective International Endometrial Tumor Analysis 4 study or who were evaluated using the same protocol. The tumors were classified according to histotype, FIGO (International Federation of Gynecology and Obstetrics) grade and FIGO stage. In addition, molecular analysis was performed for classification into the four ProMisE subtypes: polymerase‐ϵ exonuclease domain mutations (*POLE* EDM), mismatch repair proteins deficiency (MMR‐D), protein 53 wild type (p53 wt) and protein 53 abnormal (p53 abn). Demographic and preoperative sonographic characteristics, tumor recurrence or progression and survival were compared between the ProMisE subgroups. Cox regression analysis was used to identify prognostic factors associated with recurrence or progression, using univariable models to study crude associations and multivariable models to study adjusted associations. Logistic regression and receiver‐operating‐characteristics (ROC)‐curve analysis were used to assess the predictive ability of the preoperative prognostic factors regarding recurrence or progression of cancer within 3 years after surgery, and to compare their predictive ability to that of the European Society for Medical Oncology (ESMO) preoperative (based on depth of myometrial invasion, histotype and grade) and postoperative (based on histotype, grade, surgical stage and lymphovascular space invasion) risk classifications. In a separate subanalysis, cases were stratified according to ProMisE p53 abn status (present vs absent) and sonographic tumor size (anteroposterior (AP) diameter < 2 cm vs ≥ 2 cm).

**Results:**

Median follow‐up time from surgery was 58 months (interquartile range, 48–71 months; range, 0–102 months). Recurrence or progression of cancer occurred in 51/339 (15%) women, comprising 14% of those with MMR‐D, 8% of those with *POLE* EDM, 9% of those with p53 wt and 45% of those with p53 abn ProMisE subtype. On multivariable analysis, age, waist circumference, ProMisE subtype and tumor extension and AP diameter on ultrasound were associated with tumor recurrence or progression. A multivariable model comprising ProMisE subtype, age, waist circumference and sonographic tumor extension and size (area under the ROC curve (AUC), 0.89 (95% CI, 0.85–0.93)) had comparable ability to predict tumor recurrence/progression to that of a multivariable model comprising histotype, grade, age, waist circumference and sonographic tumor extension and size (AUC, 0.88 (95% CI, 0.83–0.92)), and better predictive ability than both the preoperative (AUC, 0.74 (95% CI, 0.67–0.82); *P* < 0.01) and postoperative (AUC, 0.79 (95% CI, 0.72–0.86); *P* < 0.01) ESMO risk classifications. Women with a combination of non‐p53 abn subtype and tumor size < 2 cm (164/339 (48%)) had a very low risk (1.8%) of tumor recurrence or progression.

**Conclusions:**

The combination of demographic characteristics, sonographic findings and ProMisE subtype had better preoperative predictive ability for tumor recurrence or progression than did the ESMO classification, supporting their use in the preoperative risk stratification of women with endometrial cancer. The combination of p53 status with ultrasound tumor size has the potential to identify preoperatively a large group of women with a very low risk of recurrence or progression. © 2020 The Authors. Ultrasound in Obstetrics & Gynecology published by John Wiley & Sons Ltd on behalf of International Society of Ultrasound in Obstetrics and Gynecology. ‐ Legal Statement: This is an open access article under the terms of the Creative Commons Attribution License, which permits use, distribution and reproduction in any medium, provided the original work is properly cited.

**CONTRIBUTION**

**What are the novel findings of this work?**

The combination of demographic variables (age, waist circumference), sonographic variables (tumor size and extension) and the Proactive Molecular Risk Classifier for Endometrial cancer (ProMisE) classification had better ability than the European Society for Medical Oncology (ESMO) risk classification to predict preoperatively tumor recurrence or progression in women who underwent surgery for endometrial cancer. Ultrasound tumor size < 2 cm combined with absence of protein 53 abnormal (p53 abn) ProMisE subtype can identify a large group (∼50%) of women at very low risk of recurrence or progression of disease.

**What are the clinical implications of this work?**

In women with endometrial cancer, preoperative ultrasound assessment has an independent prognostic role beyond that of the preoperative ESMO classification, as sonographic tumor size combined with p53 status can identify a large group of women with an excellent prognosis, in whom sentinel‐node biopsy or adjuvant treatment may not be considered necessary. Our findings support the use of the ProMisE molecular classification in the preoperative risk stratification of women with endometrial cancer.

## INTRODUCTION

In patients with endometrial cancer, transvaginal ultrasound can be used in conjunction with tumor histotype and grade from endometrial biopsy to predict preoperatively the risk of lymph‐node metastasis according to the European Society for Medical Oncology (ESMO), European Society for Radiotherapy & Oncology (ESTRO) and European Society of Gynaecological Oncology (ESGO) classification (referred to as ‘ESMO classification’ in this paper, for brevity)^1^. The preoperative ESMO risk classification (based on depth of myometrial invasion on imaging and tumor histotype and grade from preoperative biopsy) guides decision‐making for lymphadenectomy based on the risk for lymph‐node metastasis, whereas the postoperative ESMO risk classification (based on surgical stage, grade, histotype and lymphovascular space invasion (LVSI) from the surgical specimen) guides the use of adjuvant therapy based on the risk of recurrence.

Ultrasound is an established modality in the preoperative risk assessment for lymph‐node metastasis^1^, while the ability of ultrasound to predict before surgery recurrence or progression has not been studied. Moreover, the value of biometric variables in the prediction of adverse prognosis in women with endometrial cancer needs to be further explored.

Tumor histotype and grade are important in both the pre‐ and postoperative ESMO classification, but have limited reproducibility, particularly in high‐grade tumors^2^, ^3^, ^4^, ^5^. Moreover, agreement on tumor grade between endometrial biopsy and the hysterectomy specimen is only moderate^6^. These limitations hinder a reproducible categorization of endometrial cancer and limit the value of tumor histotype and grade as risk predictors.

The Cancer Genome Atlas (TCGA) Research Network developed a genomic classification of endometrial cancer into four prognostic subgroups (polymerase‐ϵ (*POLE*) ultramutated, microsatellite instability hypermutated, copy number low and copy number high^7^), which, however, requires costly and complex methodologies, making the TCGA classification not yet fit for clinical use. The Proactive Molecular Risk Classifier for Endometrial cancer (ProMisE) was developed and validated as a clinically applicable surrogate molecular classifier^8^, ^9^, ^10^, ^11^, rendering four corresponding prognostic subgroups: *POLE* exonuclease domain mutations (*POLE* EDM), mismatch repair proteins deficiency (MMR‐D), protein 53 wild type (p53 wt) and protein 53 abnormal (p53 abn). A molecular classification system is more robust and objective than tumor histotype and grade, as it is based on the presence or absence of a protein or mutation. It allows classification of all endometrial cancers in the preoperative setting, when several prognostic factors, such as surgical tumor stage and LVSI, are not available. Moreover, in contrast to histotype and grade, ProMisE classification on diagnostic endometrial biopsy has been shown to be highly concordant with subsequent classification on the hysterectomy specimen^11^, ^12^.

The objective of this study was to evaluate the ability of demographic and sonographic variables and the ProMisE classification to predict preoperatively tumor recurrence or progression in women with endometrial cancer.

## METHODS

The study cohort consisted of women with endometrial cancer from the Stockholm center, Karolinska University Hospital, who were part of the prospective International Endometrial Tumor Analysis (IETA) 4 study^13^ or were evaluated using the same protocol. Inclusion lasted from 1 January 2011 to 31 December 2015. End of follow‐up was on 31 August 2019. The inclusion criterion was histologically confirmed endometrial cancer based on the preoperative biopsy and/or the hysterectomy specimen. Only women with epithelial malignant tumors (endometrial carcinoma: endometrioid, mucinous, serous, clear cell, mixed cell and undifferentiated carcinomas) or mixed epithelial and mesenchymal malignant tumors (carcinosarcomas) were included. Exclusion criteria were: hysterectomy not performed or carried out at another hospital or performed more than 120 days after the ultrasound examination; final diagnosis other than endometrial cancer; incomplete ultrasound data; duplicate entries; error in the identification key; and insufficient material for the construction of a tissue microarray and isolation of genomic DNA from formalin‐fixed paraffin‐embedded tumor tissue.

The study was approved by the local ethics committee (LU 2016/362). Permission for biobanking of tissue samples was granted by the regional biobank review board (2018‐00479). All women gave written consent for use of their biobank‐stored tissue for research purposes.

All women underwent preoperative ultrasound examination by the same ultrasound expert (E.E.). Tumor size (anteroposterior (AP) tumor diameter), extension and morphology were assessed on ultrasound according to the IETA examination technique and terminology^14^. Tumor AP diameter was measured in the sagittal plane. Color/power Doppler examinations were carried out at a pulse repetition frequency of 0.3–0.9 kHz. All women underwent hysterectomy and bilateral salpingo‐oophorectomy with or without lymphadenectomy. Pathologists classified tumor stage according to the FIGO (International Federation of Gynecology and Obstetrics) 2009 staging system^15^ and tumor grade according to the FIGO grading system^16^. Detailed medical and reproductive history, using a standardized questionnaire, and biometric data (height, weight and waist circumference, measured by the physician/nurse on the day of the ultrasound examination) were included in the study protocol. All demographic and sonographic data were entered into internet‐based data capture software (Clinical Data Miner (https://cdm.esat.kuleuven.be))^17^ on the day of the ultrasound examination. Results regarding histological outcome and tumor stage were entered into the database after surgery. Data on recurrence or progression of cancer and survival were obtained through review of the patient's digital medical records.

Molecular analysis for classification into the four ProMisE subtypes (MMR‐D, *POLE* EDM, p53 wt and p53 abn) was performed retrospectively using biobank‐stored tissue from the hysterectomy specimen. Two pathologists (J.W.C., D.N.), blinded to the patient characteristics and outcomes, independently reviewed all immunohistochemistry stains, and any interpretative discrepancies were resolved by consensus, using a multiheaded microscope. As p53 immunohistochemistry staining was performed as part of routine clinical practice in all cases with endometrial cancer, it was obtained from full tumor sections. Immunohistochemistry results for p53 were categorized into three groups: Group 0, completely negative staining; Group 1, 1–80% of the tumor nuclei showed heterogeneous staining; and Group 2, > 80% of tumor nuclei showed strong positive staining. Group 1 was considered as p53 wt and Groups 0 and 2 as p53 abn. Mismatch repair (MMR) status was analyzed by immunohistochemistry for the presence of the microsatellite‐stability proteins *MSH2*, *MSH6*, *PMS2* and *MLH1* on tissue microarrays from formalin‐fixed paraffin‐embedded tumor tissue. MMR proteins were considered absent (MMR‐D) if one or more of the four microsatellite‐stability proteins were missing, or intact if all four microsatellite‐stability proteins were present. *POLE* EDM status was analyzed from genomic DNA, which was isolated from two 1‐mm core punches of formalin‐fixed paraffin‐embedded tissue. Mutations of the *POLE* gene (NM.006231) exons 9 to 14 were analyzed by Sanger sequencing. The following *POLE* mutations were considered pathogenic: *P286R*, *V411L*, *S297F*, *A456P* and *S459F*. These are the five most common pathogenic variants described for which there are strong data linking them to the ultramutated phenotypes^8^, ^18^, ^19^, ^20^. ProMisE classification of the tumors was performed according to the pragmatic model of Talhouk *et al*.^10^. According to the ProMisE decision tree, tumors were assigned to a specific molecular subtype in the following order: first, tumors with MMR proteins deficiency were classified as ‘MMR‐D’; of the remaining cases, tumors with EDM in the *POLE* gene were classified as ‘*POLE* EDM’; of the remaining cases, tumors with p53 wild type were classified as ‘p53 wt’ and the rest of the tumors with p53 null or missense mutations were classified as ‘p53 abn’.

Endometrial biopsies, through simple biopsy, dilatation and curettage or hysteroscopic resection, were performed before study inclusion and analyzed in various pathology departments in Stockholm, whereas the hysterectomy specimens were analyzed in the same department at the Karolinska University Hospital where the surgery took place. For practical reasons (i.e. access to tissue blocks), the ProMisE molecular analysis was performed on tumor tissue from the hysterectomy specimen, but the classification was used as a proxy for the preoperative biopsy specimen throughout the study, as agreement in ProMisE classification between the diagnostic biopsy and hysterectomy is high^11^, ^12^, ^21^.

The preoperative ESMO risk classification was based on findings on endometrial biopsy and transvaginal ultrasound (depth of myometrial invasion, histotype and grade) and comprised the following risk categories: low risk, Grade 1–2 endometrioid cancer without deep myometrial invasion; intermediate risk, deep myometrial invasion or Grade 3 endometrioid cancer without deep myometrial invasion; and high risk, Grade 3 endometrioid cancer with deep myometrial invasion or non‐endometrioid cancer^1^. Cases with cervical stromal invasion and extrauterine spread were included in the high‐risk group. The postoperative ESMO risk classification was based on assessment of the surgical specimen (histotype, grade, surgical stage and presence of LVSI) and cases were categorized into the following risk groups: low, intermediate, high‐intermediate, high, advanced and metastatic^1^. The advanced and metastatic risk groups were combined owing to the low number of women in these two groups.

Recurrence was defined as recurring tumor in a woman who had been tumor‐free either directly after surgery or at the end of primary treatment. The date of recurrence was defined as the date of biopsy‐confirmed recurrence in all cases but three, in which confirmative biopsy was not performed initially or not performed at all. In these three cases, recurrence date was defined as the date of recurrence according to computed tomography or clinical examination. Progression was defined as tumor progression in a woman who had remaining tumor at the end of the primary treatment. The date of progression was defined as the date of progression diagnosed by computed tomography. Overall survival was defined as time from surgery until death due to any cause, loss to follow‐up or end of follow‐up, whichever occurred first. Disease‐free survival time was defined as the time from surgery to detection of recurrence, loss to follow‐up, death due to any cause or end of follow‐up, whichever occurred first.

### Statistical analysis

Statistical analysis was performed using IBM SPSS Statistics for Windows, version 26.0 (IBM Corp., Armonk, NY, USA), STATA/IC version 12.1 (Stata Corp., College Station, TX, USA) and R version 3.6.1 (https://www.r‐project.org/). Fisher's exact test was used for comparison of categorical variables, and the Mann–Whitney *U*‐test and the Kruskal–Wallis test were used for comparison of continuous variables between two groups and between more than two groups, respectively. Categorizations for age^13^, body mass index (BMI)^22^, waist circumference^23^ and ultrasound tumor size^24^ on univariable and multivariable analyses were based on previous publications.

Demographic and sonographic characteristics, recurrence or progression of cancer and survival were compared between the ProMisE subgroups, with focus on the p53 abn subtype, which is known to be associated with adverse outcome^8^, ^9^, ^10^, ^12^, ^20^. Women were also stratified according to p53 status (present *vs* absent) and ultrasound tumor size (AP diameter < 2 cm *vs* ≥ 2 cm), which are clinically easily obtained prognostic factors known to be associated with adverse outcome^24^, ^25^, and compared regarding risk of recurrence or progression.

Survival analysis was performed using Kaplan–Meier curves, with pairwise comparison of ProMisE subtypes using the log‐rank test. Cox regression analysis was used to identify preoperative variables associated with recurrence or progression of endometrial cancer. Univariable models were used to study crude associations and multivariable models to study adjusted associations. All variables found to be significant in the univariable models were analyzed in multivariable models. The variables histotype/grade and ProMisE subtype were correlated strongly on multivariable analysis. Therefore, they were analyzed in separate, otherwise identical, multivariable models: (1) the ‘histotype‐and‐grade model’, which included tumor histotype and grade, age, waist circumference, ultrasound tumor extension and ultrasound tumor size; and (2) the ‘ProMisE model’, which included ProMisE subtype, age, waist circumference, ultrasound tumor extension and ultrasound tumor size. The ProMisE model was also adjusted for the ESMO postoperative classification, to determine if the variables had an independent association with tumor recurrence or progression, beyond the ESMO classification. All women were followed up until recurrence or progression (event), or censured owing to death, loss to follow‐up or end of follow‐up, whichever occurred first.

To evaluate whether preoperative prognostic factors associated with progression or recurrence on multivariable Cox regression analysis also had a predictive value, the histotype‐and‐grade model, the ProMisE model and the preoperative and postoperative ESMO risk classifications were analyzed using logistic regression with a fixed time (recurrence or progression within 3 years (yes/no), as all women underwent surgery at least 42 months before the study end). Their ability to predict recurrence or regression was assessed using the areas under receiver‐operating‐characteristics (ROC) curves (AUC). The statistical significance of a difference in AUC was determined using pairwise comparison through the DeLong test. These analyses were performed to assess the ability of the preoperative prognostic factors evaluated to predict recurrence or progression of endometrial cancer, and to compare their predictive ability with that of the ESMO pre‐ and postoperative risk classifications. All tests were two‐sided, and *P* < 0.05 was considered to indicate statistical significance.

## RESULTS

Eligible for inclusion were 409 women from the Stockholm center at Karolinska University Hospital who were part of the prospective IETA 4 cohort, and an additional two women who were examined at the Stockholm center according to the same protocol (*n* = 411). Of these, 72 women were excluded (underwent surgery in another hospital (*n* = 6), insufficient or no remaining tumor sample for analysis (*n* = 38), incorrect personal security number (*n* = 2), incomplete ProMisE analysis (*n* = 8), ProMisE analysis not performed (*n* = 17), duplicate case (*n* = 1)), leaving a study cohort of 339 women. Their demographic, sonographic and histopathological characteristics are presented in Table 1.

**Table 1:** Demographic, sonographic and surgical characteristics and survival data of 339 women with endometrial cancer, overall and according to whether they had tumor recurrence or progression

		Tumor recurrence or progression		
Characteristic	All (*n* = 339)	No (*n* = 288)	Yes (*n* = 51)	*P* *
Demographic				
Age (years)	67 (60–72)	66 (59–72)	70 (66–75)	< 0.01
Body mass index (kg/m^2^)	27.3 (23.5–33.0)	27.0 (23.3–33.0)	29.1 (24.6–33.1)	0.17
Waist circumference (cm)	95 (85–110)	93 (84–110)	105 (89–115)	0.02
Hypertension	170 (50.1)	138 (47.9)	32 (62.7)	0.07
Nulliparous	80 (23.6)	69 (24.0)	11 (21.6)	0.86
Postmenopausal	310 (91.4)	260 (90.3)	50 (98.0)	0.10
Use of HRT or local estrogens	82 (24.2)	69 (24.0)	13 (25.5)	0.86
Sonographic				
Tumor extension				< 0.01
MI < 50%, no CSI	221 (65.2)	205 (71.2)	16 (31.4)	
MI ≥ 50%, no CSI	69 (20.4)	53 (18.4)	16 (31.4)	
CSI with or without MI ≥ 50%	33 (9.7)	23 (8.0)	10 (19.6)	
Extrauterine spread	16 (4.7)	7 (2.4)	9 (17.6)	
Tumor AP diameter ≥ 2 cm†	137/317 (43.2)	97/269 (36.1)	40/48 (83.3)	< 0.01
Color score 3 or 4‡	214/332 (64.5)	175/282 (62.1)	39/50 (78.0)	0.04
Surgical				
Histotype				< 0.01
Endometrioid	290 (85.5)	259 (89.9)	31 (60.8)	
Non‐endometrioid	49 (14.5)	29 (10.1)	20 (39.2)	
Grade				0.03
1	141 (41.6)	132 (45.8)	9 (17.6)	
2	103 (30.4)	90 (31.3)	13 (25.5)	
3	46 (13.6)	37 (12.8)	9 (17.6)	
Stage				< 0.01
IA	205 (60.5)	195 (67.7)	10 (19.6)	
IB	72 (21.2)	55 (19.1)	17 (33.3)	
II	28 (8.3)	23 (8.0)	5 (9.8)	
III	24 (7.1)	15 (5.2)	9 (17.6)	
IV	10 (2.9)	0 (0)	10 (19.6)	
ProMisE subtype				< 0.01
MMR‐D	118 (34.8)	102 (35.4)	16 (31.4)	
*POLE* EDM	26 (7.7)	24 (8.3)	2 (3.9)	
p53 wt	151 (44.5)	138 (47.9)	13 (25.5)	
p53 abn	44 (13.0)	24 (8.3)	20 (39.2)	
Adjuvant therapy	113 (33.3)	81 (28.1)	32 (62.7)	< 0.01
Survival data				
Death from disease	32 (9.4)	0 (0)	32 (62.7)	< 0.01
Death from other/unknown cause	16 (4.7)	16 (5.6)	0 (0)	
5‐year overall survival (%)§	87	96	38	< 0.01

Data are presented as median (interquartile range), *n* (%) or *n*/*N* (%), unless indicated otherwise.

* Comparison using Mann–Whitney *U*‐test for continuous variables and Fisher's exact test for categorical variables.

† In 317 women with defined tumor on ultrasound.

‡ In 332 women with visible endometrium on ultrasound.

§ Estimated from Kaplan–Meier curves using log‐rank test.

AP, anteroposterior; CSI, cervical stromal invasion; HRT, hormone replacement therapy; MI, myometrial invasion; MMR‐D, mismatch repair proteins deficiency; p53 abn, protein 53 abnormal; p53 wt, protein 53 wild type; *POLE* EDM, polymerase‐ϵ exonuclease domain mutations; ProMisE, Proactive Molecular Risk Classifier for Endometrial cancer.

Lymphadenectomy was performed in 91 (27%) women, of whom 21 (23%) had lymph‐node metastasis. Median follow‐up time from surgery was 58 months (interquartile range, 48–71 months; range, 0–102 months). Two women died within 1 month after surgery: one after 30 days owing to postoperative complications and the other of unknown cause in her home after 3 days. Three women were lost to follow‐up after 29, 30 and 40 months because they moved to another county. Overall, 51 (15%) women had tumor recurrence or progression. This was detected because of symptoms in half of the women and at routine follow‐up in the other half. The vast majority (38/43 (88%)) of recurrences occurred within 3 years and all progressions (100% (8/8)) within 2 years after surgery. Women with tumor recurrence or progression, compared with those without, were significantly older and had a larger waist circumference, their tumors were more often non‐endometrioid, of ProMisE subtype p53 abn and of higher stage, and on ultrasound their tumors were larger, with a higher color score and more advanced tumor extension (Table 1).

**Figure 1:**
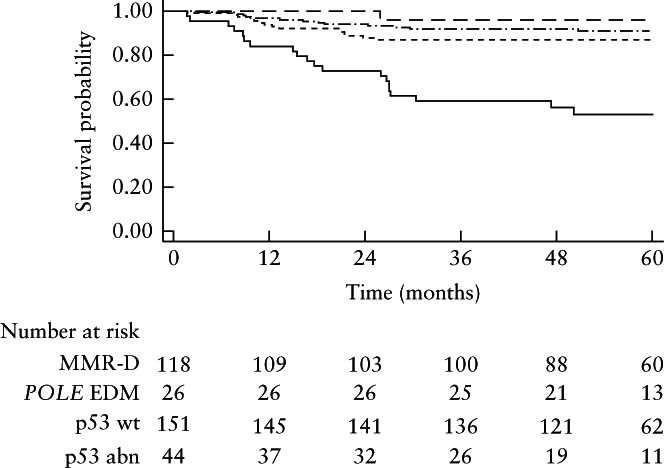
Kaplan–Meier plot of probability of tumor recurrence or progression after surgery in 339 women with endometrial cancer, according to Proactive Molecular Risk Classifier for Endometrial cancer subtype: mismatch repair proteins deficiency (MMR‐D; 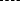), polymerase‐ϵ exonuclease domain mutations (*POLE* EDM; 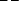), protein 53 wild type (p53 wt; 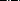) and protein 53 abnormal (p53 abn; 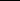).

The clinical, sonographic and histopathological characteristics of the women according to their ProMisE subtype are presented in Table 2. Compared with the other subtypes, p53 abn was associated with older age, larger tumors on preoperative ultrasound, non‐endometrioid cancer, higher cancer stage, more advanced postoperative ESMO risk group, higher likelihood of death from disease and lower 5‐year disease‐free survival and overall survival. A Kaplan–Meier plot on tumor recurrence or progression according to ProMisE subtype is presented in Figure 1, and shows that women with p53 abn had a higher probability of recurrence or progression than did the other three subgroups.

**Table 2:** Demographic, sonographic and surgical characteristics and survival data of 339 women with endometrial cancer, according to Proactive Molecular Risk Classifier for Endometrial cancer (ProMisE) subtype

Characteristic	MMR‐D (*n* = 118)	*POLE* EDM (*n* = 26)	p53 wt (*n* = 151)	p53 abn (*n* = 44)	*P* *	*P* †
Demographic						
Age (years)	68 (61–72)	62 (52–65)	67 (60–71)	70 (64–75)	< 0.01	0.04
Body mass index (kg/m^2^)	27.3 (24.0–33.0)	25.1 (22.0–31.9)	27.5 (23.6–35.0)	26.9 (23.6–30.7)	0.24	0.47
Waist circumference (cm)	95 (85–110)	87 (83–103)	97 (84–115)	95 (86–107)	0.33	0.76
Sonographic						
Visible endometrium	118 (100)	26 (100)	146 (96.7)	42 (95.5)		
Endometrial–myometrial junction					0.14	0.22
Regular	23 (19.5)	2 (7.7)	35/146 (24.0)	5/42 (11.9)		
Irregular/interrupted/undefined	95 (80.5)	24 (92.3)	111/146 (76.0)	37/42 (88.1)		
Endometrial morphology					< 0.01	0.40
Uniform	66 (55.9)	13 (50.0)	107/146 (73.3)	24/42 (57.1)		
Non‐uniform	52 (44.1)	13 (50.0)	39/146 (26.7)	18/42 (42.9)		
Color score					0.01	0.06
1 or 2 (no or minimal flow)	34 (28.8)	10 (38.5)	65/146 (44.5)	9/42 (21.4)		
3 or 4 (moderate or abundant flow)	84 (71.2)	16 (61.5)	81/146 (55.5)	33/42 (78.6)		
Vascular pattern					0.96	0.62
Multiple vessels with multifocal origin	50 (42.4)	10 (38.5)	63/146 (43.2)	20/42 (47.6)		
Other	68 (57.6)	16 (61.5)	83/146 (56.8)	22/42 (52.4)		
Tumor defined	114 (96.6)	26 (100)	138 (91.4)	39 (88.6)		
Tumor AP diameter (mm)	20.0 (13.0–27.0)	13.5 (9.0–24.0)	14.0 (9.8–25.0)	26.0 (14.0–36.0)	< 0.01	< 0.01
Surgical						
Histotype					< 0.01	< 0.01
Endometrioid	105 (89.0)	26 (100)	149 (98.7)	10 (22.7)		
Non‐endometrioid	13 (11.0)	0 (0)	2 (1.3)	34 (77.3)		
Grade					< 0.01	< 0.01
1	36 (30.5)	11 (42.3)	93 (61.6)	1 (2.3)		
2	51 (43.2)	5 (19.2)	45 (29.8)	2 (4.5)		
3	18 (15.3)	10 (38.5)	11 (7.3)	7 (15.9)		
Stage					< 0.01	< 0.01
I	97 (82.2)	23 (88.5)	131 (86.8)	26 (59.1)		
II–IV	21 (17.8)	3 (11.5)	20 (13.2)	18 (40.9)		
Postoperative ESMO risk group‡					< 0.01	< 0.01
Low	54 (45.8)	11 (42.3)	89 (58.9)	3 (6.8)		
Intermediate	10 (8.5)	1 (3.8)	26 (17.2)	0 (0)		
High‐intermediate	17 (14.4)	7 (26.9)	15 (9.9)	2 (4.5)		
High	35 (29.7)	7 (26.9)	17 (11.3)	33 (75.0)		
Advanced or metastatic	2 (1.7)	0 (0)	4 (2.6)	6 (13.6)		
Survival data						
Recurrence or progression	16 (13.6)	2 (7.7)	13 (8.6)	20 (45.5)	< 0.01	< 0.01
Death from disease	7 (5.9)	1 (3.8)	7 (4.6)	17 (38.6)	< 0.01	< 0.01
5‐year disease‐free survival (%)§	83	96	87	51	< 0.01	< 0.01
5‐year overall survival (%)§	90	96	91	58	< 0.01	< 0.01

Data are presented as median (interquartile range), *n* (%) or *n*/*N* (%), unless indicated otherwise.

* Comparison of four subgroups using Kruskal–Wallis test for continuous variables and Fisher's exact test for categorical variables.

† Comparison of p53 abn subgroup *vs* other three subgroups using Mann–Whitney *U*‐test for continuous variables and Fisher's exact test for categorical variables.

‡ Based on postoperative European Society for Medical Oncology (ESMO) risk classification^1^.

§ Estimated from Kaplan–Meier plots using log‐rank test.

AP, anteroposterior; MMR‐D, mismatch repair proteins deficiency; p53 abn, protein 53 abnormal; p53 wt, protein 53 wild type; *POLE* EDM, polymerase‐ϵ exonuclease domain mutations.

All evaluated preoperative variables apart from BMI were associated with tumor recurrence or progression on univariable analysis (Table 3). Among the ProMisE subtypes, only p53 abn was associated with recurrence or progression. On multivariable analysis (which included all preoperative variables significantly associated with tumor recurrence or progression on univariable analysis) only age, waist circumference, ProMisE subtype, tumor extension on ultrasound and tumor AP diameter on ultrasound remained associated with tumor recurrence or progression (Table 4).

**Table 3:** Univariable Cox regression analysis for association of preoperative variables with tumor recurrence or progression in 339 women with endometrial cancer

Variable	*n*	Recurrence or progression (*n* (%))	Hazard ratio (95% CI)	*P* *
Demographic				
Age				< 0.01
< 65 years	131	8 (6.1)	Reference	
≥ 65 years	208	43 (20.7)	3.7 (1.7–7.8)	
Body mass index				0.1
< 25 kg/m^2^	117	13 (11.1)	Reference	
≥ 25 kg/m^2^	222	38 (17.1)	1.6 (0.8–3.0)	
Waist circumference				0.03
< 88 cm	120	11 (9.2)	Reference	
≥ 88 cm	219	40 (18.3)	2.1 (1.1–4.0)	
Histopathological				< 0.01
Histotype and grade on biopsy				
Endometrioid, Grade 1 or 2	229	21 (9.2)	Reference	
Endometrioid, Grade 3	32	8 (25.0)	3.0 (1.3–6.7)	
Non‐endometrioid	48	20 (41.7)	5.3 (2.9–9.8)	
Other†	30	2 (6.7)	0.7 (0.2–3.1)	
ProMisE subtype‡				< 0.01
p53 wt	151	13 (8.6)	Reference	
MMR‐D	118	16 (13.6)	1.6 (0.8–3.4)	
*POLE* EDM	26	2 (7.7)	0.9 (0.2–3.8)	
p53 abn	44	20 (45.5)	6.5 (3.2–13.2)	
Sonographic				
Tumor extension				< 0.01
MI < 50%, no CSI	221	16 (7.2)	Reference	
MI ≥ 50%, no CSI	69	16 (23.2)	3.4 (1.7–6.9)	
CSI with or without MI ≥ 50%	33	10 (30.3)	4.9 (2.2–10.7)	
Extrauterine spread	16	9 (56.3)	11.4 (5.0–25.8)	
Tumor AP diameter				< 0.01
< 2 cm	180	8 (4.4)	Reference	
≥ 2 cm	137	40 (29.2)	7.8 (3.7–16.8)	
Tumor not defined	22	3 (13.6)	3.4 (0.9–12.7)	
Endometrial–myometrial junction§				0.01
Regular	65	3 (4.6)	Reference	
Irregular/interrupted/undefined	267	47 (17.6)	4.3 (1.3–13.8)	
Endometrial morphology§				0.03
Uniform	210	25 (11.9)	Reference	
Non‐uniform	122	25 (20.5)	1.8 (1.05–3.2)	
Color score§				0.04
1 or 2 (no or minimal flow)	118	11 (9.3)	Reference	
3 or 4 (moderate or abundant flow)	214	39 (18.2)	2.1 (1.1–4.0)	
Vascular pattern§				< 0.01
Other	189	18 (9.5)	Reference	
Multiple multifocal	143	32 (22.4)	2.5 (1.4–4.5)	
Preoperative ESMO risk group¶				< 0.01
Low	164	8 (4.9)	Reference	
Intermediate	49	10 (20.4)	4.4 (1.7–11.1)	
High	96	31 (32.3)	7.6 (3.5–16.6)	
Other†	30	2 (6.7)	1.4 (0.3–6.5)	

* Test of variable including all categories.

† Endometrioid cancer not graded (*n* = 5), suspicion of endometrial cancer (*n* = 24), no biopsy (*n* = 1).

‡ Analyzed on hysterectomy specimen but used as proxy for preoperative biopsy.

§ In 332 cases with visible endometrium on ultrasound.

¶ Based on preoperative European Society for Medical Oncology (ESMO) risk classification^1^.

AP, anteroposterior; CSI, cervical stromal invasion; MI, myometrial invasion; MMR‐D, mismatch repair proteins deficiency; p53 abn, protein 53 abnormal; p53 wt, protein 53 wild type; *POLE* EDM, polymerase‐ϵ exonuclease domain mutations; ProMisE, Proactive Molecular Risk Classifier for Endometrial cancer.

**Table 4:** Multivariable Cox regression analysis for association of preoperative variables with tumor recurrence or progression in 339 women with endometrial cancer

	All variables significant on univariable analysis		Histotype‐and‐grade model*		ProMisE model†		ProMisE model adjusted for ESMO post†	
Variable	HR (95% CI)	*P* ‡	HR (95% CI)	*P* ‡	HR (95% CI)	*P* ‡	HR (95% CI)	*P* ‡
Demographic								
Age		< 0.01		< 0.01		< 0.01		< 0.01
< 65 years	Reference		Reference		Reference		Reference	
≥ 65 years	4.0 (1.7–9.5)		4.4 (2.0–9.8)		3.8 (1.7–8.4)		4.1 (1.7–9.5)	
Waist circumference		0.01		0.01		0.02		0.01
< 88 cm	Reference		Reference		Reference		Reference	
≥ 88 cm	2.6 (1.2–5.6)		2.5 (1.2–5.1)		2.5 (1.2–5.1)		2.6 (1.2–5.5)	
Histopathological								
Histotype and grade on biopsy		0.40		< 0.01				
Endometrioid, Grade 1 or 2	Reference		Reference		—	—	—	—
Endometrioid, Grade 3	2.0 (0.8–5.0)		2.6 (1.1–6.0)		—		—	
Non‐endometrioid	1.9 (0.7–4.8)		4.4 (2.3–8.2)		—		—	
Other§	0.8 (0.2–3.5)		0.8 (0.2–3.4)		—		—	
ProMisE subtype¶		0.04				< 0.01		0.02
p53 wt	Reference		—	—	Reference		Reference	
MMR‐D	1.1 (0.5–2.4)		—		1.1 (0.5–2.4)		1.5 (0.7–3.4)	
*POLE* EDM	1.0 (0.2–5.1)		—		1.3 (0.3–6.3)		1.9 (0.4–9.6)	
p53 abn	3.9 (1.3–11.1)		—		5.7 (2.8–11.7)		4.6 (1.7–12.5)	
Sonographic								
Tumor extension		< 0.01		< 0.01		< 0.01		0.01
MI < 50%, no CSI	Reference		Reference		Reference		Reference	
MI ≥ 50%, no CSI	1.4 (0.5–3.5)		1.4 (0.6–3.0)		1.6 (0.7–3.5)		1.2 (0.5–2.9)	
CSI with or without MI ≥ 50%	1.8 (0.6–5.5)		2.2 (0.9–5.3)		2.2 (0.9–5.4)		2.0 (0.7–5.5)	
Extrauterine spread	9.7 (3.0–30.7)		7.4 (2.8–19.7)		11.5 (4.2–31.0)		6.5 (1.8–23.4)	
Tumor AP diameter		< 0.01		< 0.01		0.01		0.01
< 2 cm	Reference		Reference		Reference		Reference	
≥ 2 cm	4.7 (1.8–12.4)		3.9 (1.6–9.7)		3.8 (1.6–9.4)		4.3 (1.6–11.3)	
Tumor not defined	5.3 (1.02–27.2)		4.2 (1.1–16.7)		3.8 (0.96–15.3)		4.1 (1.01–16.8)	
Endometrial–myometrial junction**		0.30		—		—		—
Regular	Reference		—		—		—	
Irregular/interrupted/undefined	2.0 (0.6–7.4)		—		—		—	
Endometrial morphology**		0.30		—		—		—
Uniform	Reference		—		—		—	
Non‐uniform	1.3 (0.7–2.4)		—		—		—	
Color score**		0.07		—		—		—
1 or 2 (no or minimal flow)	Reference		—		—		—	
3 or 4 (moderate or abundant flow)	0.4 (0.2–1.1)		—		—		—	
Vascular pattern**		0.60		—		—		—
Other	Reference		—		—		—	
Multiple multifocal	1.2 (0.5–3.0)		—		—		—	

* Histotype‐and‐grade model includes histotype, grade, age, waist circumference and sonographic tumor size and extension.

† ProMisE model includes ProMisE subtype, age, waist circumference and sonographic tumor size and extension.

‡ Test of variable including all categories.

§ Endometrioid cancer not graded (*n* = 5), suspicion of endometrial cancer (*n* = 24), no biopsy (*n* = 1).

¶ Analyzed on hysterectomy specimen but used as proxy for preoperative biopsy.

** In 332 cases with visible endometrium on ultrasound.

AP, anteroposterior; CSI, cervical stromal invasion; ESMO post, postoperative European Society for Medical Oncology risk classification^1^; HR, hazard ratio; MI, myometrial invasion; MMR‐D, mismatch repair proteins deficiency; p53 abn, protein 53 abnormal; p53 wt, protein 53 wild type; *POLE* EDM, polymerase‐ϵ exonuclease domain mutations; ProMisE, Proactive Molecular Risk Classifier for Endometrial cancer.

**Figure 2:**
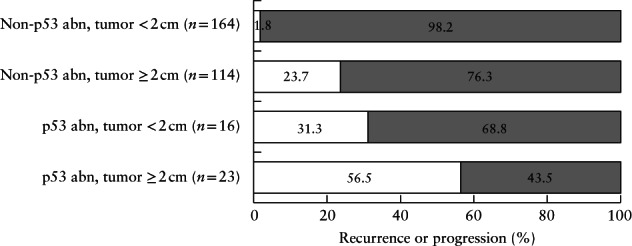
Tumor recurrence or progression in 317 women with defined tumor on preoperative ultrasound who underwent surgery for endometrial cancer, stratified according to preoperative sonographic tumor size (anteroposterior diameter < 2 cm *vs* ≥ 2 cm) and Proactive Molecular Risk Classifier for Endometrial cancer subtype protein 53 abnormal (p53 abn) status. 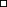, recurrence or progression; 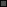, no recurrence or progression.

Tumor size on preoperative ultrasound remained associated with tumor recurrence or progression in all multivariable analyses performed (Table 4). In women with defined tumor on ultrasound (*n* = 317), tumor AP diameter ≥ 2 cm, as compared with < 2 cm, was associated with deep myometrial invasion (55% (76/137) *vs* 16% (29/180); *P* < 0.01), higher incidence of lymph‐node metastasis among those undergoing lymphadenectomy (*n* = 84) (30% (17/57) *vs* 7% (2/27); *P* = 0.03), decreased survival (5‐year overall survival, 78% *vs* 93%; *P* < 0.01) and a higher risk of tumor recurrence or progression (29% (40/137) *vs* 4% (8/180); *P* < 0.01); this was also the case among the 154 women with preoperative ESMO low risk (15% (4/27) *vs* 3% (4/127); *P* = 0.03). Stratification of women according to tumor size (AP diameter < 2 cm *vs* ≥ 2 cm) and p53 abn status (p53 abn *vs* non‐p53 abn) showed that women with the combination of AP diameter < 2 cm and non‐p53 abn status (164/339 (48%)) were at very low risk of recurrence or progression (3/164 (1.8%) (95% CI, 0.4–3.2%)) (Figure 2).

**Figure 3:**
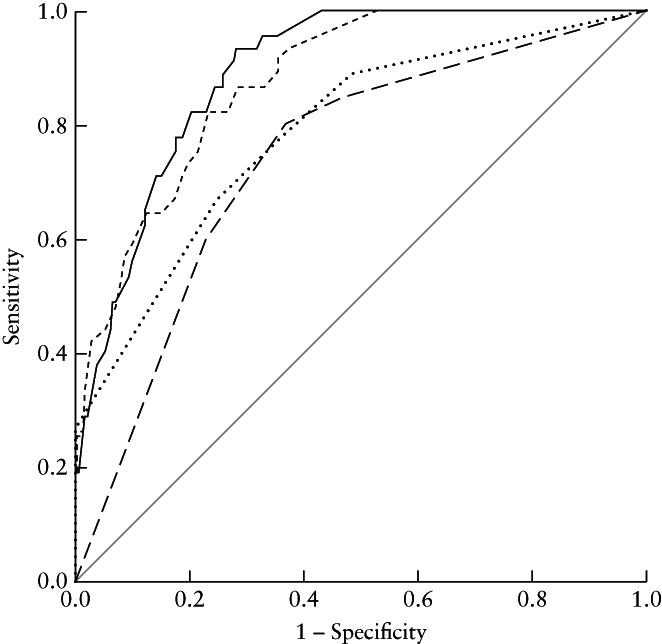
Receiver‐operating‐characteristics curves showing prediction of tumor recurrence or progression within 3 years after surgery for endometrial cancer by the Proactive Molecular Risk Classifier for Endometrial cancer (ProMisE) model (based on ProMisE subtype, age, waist circumference and sonographic tumor size and extension) (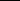), the histotype‐and‐grade model (based on tumor histotype and grade, age, waist circumference and sonographic tumor size and extension) (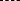), the preoperative European Society for Medical Oncology (ESMO) risk classification^1^ (based on depth of myometrial invasion, histotype and grade) (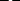) and the postoperative ESMO risk classification^1^ (based on surgical stage, grade, histotype and lymphovascular space invasion) (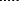).

The prognostic factors associated with tumor recurrence or progression within 3 years after surgery are presented in Table 5. These multivariable models constituted the basis for the ROC‐curve analysis comparing the ability to predict recurrence or progression of cancer within 3 years after surgery of the ProMisE model, the histotype‐and‐grade model, the preoperative ESMO risk classification and the postoperative ESMO risk classification (Figure 3). The ability of the ProMisE model to predict tumor recurrence or progression within 3 years after surgery (AUC, 0.89 (95% CI, 0.85–0.93)) was comparable with that of the histotype‐and‐grade model (AUC, 0.88 (95% CI, 0.83–0.92); *P* = 0.22) and higher than that of both the preoperative (AUC, 0.74 (95% CI, 0.67–0.82); *P* < 0.01) and postoperative (AUC, 0.79 (95% CI, 0.72–0.86); *P* < 0.01) ESMO risk classifications (Figure 3). The ProMisE model was also superior to the ProMisE classification alone (AUC, 0.70 (95% CI, 0.61–0.79); *P* < 0.01).

**Table 5:** Multivariable logistic regression analysis for association of preoperative variables with tumor recurrence or progression within 36 months after surgery in 339 women with endometrial cancer

			All variables significant on univariable analysis		Histotype‐and‐grade model*		ProMisE model†	
Predictor	*n*	Recurrence or progression (*n* (%))	OR (95% CI)	*P* ‡	OR (95% CI)	*P* ‡	OR (95% CI)	*P* *‡*
Demographic								
Age				< 0.01		< 0.01		< 0.01
< 65 years	131	7 (5.3)	Reference		Reference		Reference	
≥ 65 years	208	39 (18.8)	5.9 (2.0–17.2)		6.7 (2.3–19.4)		5.7 (2.0–16.3)	
Waist circumference				< 0.01		0.03		< 0.01
< 88 cm	120	8 (6.7)	Reference		Reference		Reference	
≥ 88 cm	219	38 (17.4)	4.0 (1.5–11.1)		4.3 (1.6–11.2)		3.9 (1.4–10.6)	
Histopathological				0.50		< 0.01		—
Histotype and grade on biopsy								
Endometrioid, Grade 1 or 2	229	19 (8.3)	Reference		Reference		—	
Endometrioid, Grade 3	32	8 (25.0)	1.9 (0.6–6.2)		3.0 (1.03–9.0)		—	
Non‐endometrioid	48	17 (35.4)	2.0 (0.5–7.8)		5.7 (2.3–14.3)		—	
Other§	30	2 (6.7)	0.6 (0.1–3.3)		0.7 (0.1–3.5)		—	
ProMisE subtype¶				0.10		—		< 0.01
p53 wt	151	12 (7.9)	Reference		—		Reference	
MMR‐D	118	15 (12.7)	1.3 (0.5–3.5)		—		1.5 (0.6–3.9)	
*POLE* EDM	26	1 (3.8)	0.7 (0.07–7.7)		—		1.0 (0.1–10.3)	
p53 abn	44	18 (40.9)	5.0 (1.2–20.9)		—		9.1 (3.3–25.5)	
Sonographic								
Tumor extension				< 0.01		0.02		< 0.01
MI < 50%, no CSI	221	14 (6.3)	Reference		Reference		Reference	
MI ≥ 50%, no CSI	69	14 (20.3)	1.6 (0.6–4.4)		1.4 (0.5–3.9)		1.7 (0.6–4.8)	
CSI with or without MI ≥ 50%	33	10 (30.3)	3.0 (0.9–10.2)		2.7 (0.8–9.0)		3.2 (0.9–10.5)	
Extrauterine spread	16	8 (50.0)	13.5 (2.9–63.4)		10.0 (2.2–45.3)		16.2 (3.5–74.8)	
Tumor AP diameter				< 0.01		< 0.01		< 0.01
< 2 cm	180	6 (3.3)	Reference		Reference		Reference	
≥ 2 cm	137	37 (27.0)	5.8 (1.9–17.8)		5.7 (1.9–17.3)		5.7 (1.8–17.4)	
Tumor not defined	22	3 (13.6)	5.9 (1.1–31.9)		6.2 (1.2–31.7)		5.8 (1.1–30.8)	
Preoperative ESMO risk group**				< 0.01				
Low	164	7 (4.3)	Reference					
Intermediate	49	9 (18.4)	5.0 (1.8–14.4)					
High	96	28 (29.2)	9.2 (3.8–22.2)					
Other§	30	2 (6.7)	1.6 (0.3–8.1)					
Postoperative ESMO risk group††				0.03				
Low	157	5 (3.2)	Reference					
Intermediate	37	5 (13.5)	4.8 (1.3–17.4)					
High‐intermediate	41	5 (12.2)	4.2 (1.2–15.4)					
High	92	19 (20.7)	7.9 (2.8–22.0)					
Advanced or metastatic	12	12 (100.0)	NA					

* Histotype‐and‐grade model includes histotype, grade, age, waist circumference and sonographic tumor size and extension.

† ProMisE model includes ProMisE subtype, age, waist circumference and sonographic tumor size and extension.

‡ Test of variable including all categories.

§ Endometrioid cancer not graded (*n* = 5), suspicion of endometrial cancer (*n* = 24), no biopsy (*n* = 1).

¶ Analyzed on hysterectomy specimen but used as proxy for preoperative biopsy.

** Based on preoperative European Society for Medical Oncology (ESMO) risk classification^1^.

†† Based on postoperative ESMO risk classification^1^.

AP, anteroposterior; CSI, cervical stromal invasion; MI, myometrial invasion; MMR‐D, mismatch repair proteins deficiency; NA, odds ratio (OR) estimate not available owing to recurrence or progression in all women; p53 abn, protein 53 abnormal; p53 wt, protein 53 wild type; *POLE* EDM, polymerase‐ϵ exonuclease domain mutations; ProMisE, Proactive Molecular Risk Classifier for Endometrial cancer.

## DISCUSSION

We have verified that the ProMisE subtype p53 abn is an adverse prognostic factor, as it was associated with larger tumors on preoperative ultrasound, non‐endometrioid cancer, higher stage, increased risk of recurrence or progression and reduced survival, compared with the other ProMisE subtypes. The combination of non‐p53 abn status and sonographic tumor AP diameter < 2 cm showed the potential to identify a large group of women (48%) at very low risk (1.8%) of recurrence or progression of disease. The ProMisE model, including ProMisE subtype and demographic (age, waist circumference) and sonographic (tumor size and extension) prognostic factors, had better predictive ability for the recurrence or progression of disease than did the current pre‐ and postoperative ESMO risk classifications, supporting its use in preoperative risk stratification.

Strengths of this study include the prospective study cohort, which represents a general population and not a high‐risk sample, recruited during a recent and limited period of time, and the comprehensive prospective cohort database containing detailed clinical information, in which all data were locked after being saved. Nearly half of the study population had a follow‐up of 5 years or longer and all had high‐quality ultrasound data.

A shortcoming of the study is that the ProMisE molecular analysis was performed on the hysterectomy specimen and not on the preoperative endometrial biopsy sample. However, it has been shown that ProMisE classification is highly concordant between preoperative biopsy and subsequent hysterectomy specimen^11^, ^12^, and it has been concluded that the results of molecular markers, such as p53, on hysterectomy specimen can be safely translated towards the preoperative endometrial biopsy^21^. Another limitation is that the prognostic factors were identified from the same dataset that was used to compare their predictive ability with that of the ESMO classification, which may have favored the ProMisE and histotype‐and‐grade models. The prognostic factors for tumor recurrence or progression identified in our study could serve as a basis for future studies aiming to create preoperative risk‐prediction models for recurrence or progression of endometrial cancer, based on a larger cohort, with ProMisE analysis performed on preoperative biopsy specimens and variable selection based on *a‐priori* knowledge and with external validation.

Previous studies have found that tumor size on hysterectomy specimen^24^, ^25^, ^26^, ultrasound^13^ and MRI^27^ are predictive of lymph‐node metastasis^24^, ^25^, ^26^, ^27^, survival^24^, ^25^ and high‐risk disease^13^. In accordance with these data, we found that tumors with an AP diameter of ≥ 2 cm on preoperative ultrasound were associated with more advanced tumor extension on ultrasound, increased likelihood of lymph‐node metastasis and worse survival outcomes, supporting the potential for sonographic tumor size to predict adverse outcome before surgery. In addition, our findings show that tumor size on preoperative ultrasound is an independent predictor of recurrence or progression of disease, even among cases classified as low risk by the preoperative ESMO classification.

Increased BMI, consistent with being overweight or obese, is associated with an increased risk of the development of endometrial cancer^28^, and obesity at diagnosis has been associated with worse survival, though there is insufficient evidence to establish an increased risk of recurrence^29^. Changes in insulin resistance, systemic inflammation and alterations in the levels of hormones and growth factors have all been implicated in promoting the development and progression of cancer in those who are overweight or obese^30^. In spite of these data, we found no association between BMI and tumor recurrence or progression (Table 3). However, a waist circumference of ≥ 88 cm was found to be an independent predictor of recurrence or progression of cancer, with the risk being more than double that of women with a waist circumference of < 88 cm (Tables 4 and 5). This discrepancy might be explained by the fact that BMI constitutes a poor proxy for adiposity, as it does not

describe the distribution of adipose tissue or distinguish adipose tissue from muscle mass^30^. In postmenopausal women with endometrial cancer, subcutaneous fat contributes more to estradiol levels than does visceral fat, indicating that subcutaneous fat might be relevant for the carcinogenesis of endometrial cancer^31^, and women with increasing visceral fat percentage have a significantly reduced disease‐specific survival, independent of BMI^32^. This indicates that the location of body fat is prognostic, and it can be stated that our divergent findings on BMI and waist circumference indicate that abdominal adiposity is a worse prognostic factor than is general adiposity. To the best of out knowledge, this is the first time that waist circumference has been reported as an independent predictor of recurrence or progression of endometrial cancer.

The ProMisE classification was validated in a population‐based cohort of 452 women^11^, which was similar to our cohort. The ProMisE subtype distribution was comparable to that in our cohort, and the study also identified p53 abn as an adverse prognostic factor. The ProMisE classification has several advantages, as it shows a higher concordance between preoperative endometrial biopsy and hysterectomy compared with tumor histotype and grade^11^, ^12^, is able to differentiate high‐grade tumors with excellent prognosis (*POLE* EDM) from tumors with poor prognosis (p53 abn)^8^, ^9^, ^10^, can evaluate tumors in the gray zone between endometrioid and serous histotype and can identify women with MMR‐D, who may have Lynch syndrome and who should be referred for genetic counseling and testing^8^, ^9^, ^10^.

In the era of sentinel‐node biopsy, the clinical importance of preoperative ultrasound has been questioned, at least in situations in which sentinel‐node biopsy is offered to all women. This study indicates, however, that ultrasound variables have an independent prognostic role beyond the ESMO risk classification and that the combination of ultrasound and p53 status, often obtained on routine histopathology assessment, can identify a large group of women (48%) at very low risk of tumor recurrence or progression (1.8%) in whom neither sentinel‐node biopsy nor adjuvant treatment may be considered necessary. Moreover, the combination of demographic characteristics, sonographic findings and ProMisE subtype had a better preoperative predictive ability for the recurrence or progression of disease than did the ESMO risk classification, supporting its use in the preoperative risk stratification of women with endometrial cancer.

## Data Availability

The data that support the findings of this study are available from the corresponding author upon reasonable request.
